# Bosentan Delivery via Nano Metal-Organic Framework nanoMIL-89 Restores Vascular Homeostasis in Pulmonary Arterial Hypertension

**DOI:** 10.2147/IJN.S535437

**Published:** 2025-09-10

**Authors:** Mashael A Al-Badr, Hanan H Abunada, Richa Gill, Hend S Fayed, Ayman Al Haj Zen, Mohammad A Al-Ghouti, Md Mizanur Rahman, Nura A Mohamed, Haissam Abou-Saleh

**Affiliations:** 1 Biomedical Research Center, QU Health, Qatar University, Doha, Qatar; 2 Biological Science Program, Department of Biological and Environmental Sciences, College of Arts and Sciences, Qatar University, Doha, Qatar; 3 Environmental Science Program, Department of Biological and Environmental Sciences, College of Arts and Sciences, Qatar University, Doha, Qatar; 4 College of Health and Life Sciences, Hamad Bin Khalifa University, Doha, Qatar; 5 Department of Biomedical Sciences, College of Health Sciences, QU Health, Qatar University, Doha, Qatar

**Keywords:** nanoparticles, nanomedicine, PAH, endothelial dysfunction, drug delivery, vascular homeostasis

## Abstract

**Background:**

Pulmonary arterial hypertension (PAH) is a progressive vascular disorder characterized by endothelial dysfunction, smooth muscle proliferation, and inflammation. Current treatments, such as Bosentan (an endothelin receptor antagonist), are limited by systemic toxicity and a short half-life. This study aimed to evaluate a nanomedicine formulation of Bosentan using the iron-based metal–organic framework MIL-89 (nanoMIL-89) as a targeted drug delivery platform.

**Methods:**

Bosentan-loaded nanoMIL-89 (Bosentan@nanoMIL-89) was synthesized and characterized using microscopy, XRD, FTIR, and HPLC. In vitro assays were conducted on human umbilical vein endothelial cells (HUVECs) and human pulmonary artery smooth muscle cells (HPASMCs) under both basal and lipopolysaccharide (LPS)-induced inflammatory conditions.

**Results:**

Bosentan@nanoMIL-89 exhibited no significant cytotoxic or genotoxic effects while maintaining cellular viability. Under basal conditions, it reduced CXCL8 expression by up to 64.38% in HUVECs and 43.34% in HPASMCs. In lipopolysaccharide (LPS)-induced inflammatory conditions, CXCL8 suppression was further enhanced to 94.20% in HUVECs and 58.14% in HPASMCs. In HUVECs, Bosentan@nanoMIL-89 also decreased endothelin-1 (ET-1) release by up to 96.68% and reduced reactive oxygen species (ROS) levels by 46.17% under non-inflammatory conditions. These dose-dependent effects underscore its potent anti-inflammatory and antioxidant properties. Furthermore, Bosentan@nanoMIL-89 promoted angiogenic activity in HUVECs, suggesting therapeutic potential for vascular repair.

**Conclusion:**

These findings highlight Bosentan@nanoMIL-89 as a promising nanotherapeutic platform for PAH. By improving efficacy while mitigating systemic side effects, this approach reinforces the broader potential of MOF-based drug delivery systems in the management of vascular diseases.

## Graphical Abstract

**Figure:**
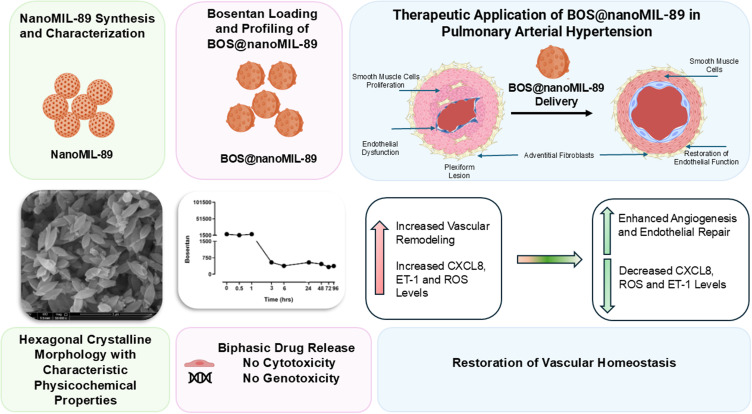

## Introduction

PAH is a complex disease characterized by increased pulmonary vascular resistance, vasoconstriction, endothelial dysfunction, and vascular remodeling.^1^ Due to non-specific and overlapping symptoms with other cardiopulmonary diseases, PAH is often diagnosed later in the disease course, thereby delaying treatment and increasing the risk of right heart failure and mortality.^2^,^3^ The condition is defined by a mean pulmonary artery pressure greater than 20 mmHg, with typical features including an imbalance between vasoconstrictor molecules, such as endothelin-1, and vasodilators like nitric oxide and prostacyclin, enhanced proliferation of HPASMCs, and the formation of plexiform lesions that block pulmonary blood flow.^4^,^5^

Despite the availability of current treatments for pulmonary arterial hypertension (PAH)—including endothelin receptor antagonists (eg, Bosentan), phosphodiesterase-5 inhibitors (eg, sildenafil), and prostacyclin analogues—their clinical effectiveness is often constrained by short half-lives, systemic side effects, and suboptimal pharmacokinetics.^6–8^ Among these, Bosentan, a dual ETA/ETB receptor antagonist, has become a cornerstone therapy for PAH, with a half-life of 5–8 hours.^8^ Its molecular formula (C_27_H_29_N_5_O₆S) and standard dosing regimen (initially 62.5 mg twice daily, marketed as Tracleer) are well established.^9^ However, Bosentan therapy is frequently associated with adverse effects such as blurred vision, dizziness, confusion, and loss of appetite. These limitations underscore the urgent need for advanced drug delivery systems capable of improving targeting, controlled release, and overall bioavailability.

Nanomedicine, particularly metal-organic frameworks (MOFs), presents a promising approach for advanced pulmonary drug delivery. MOFs are porous crystalline hybrid materials formed by metal ions and organic linkers, capable of encapsulating diverse therapeutic agents with controlled release properties.^10^ Among these, iron-based MIL-type nanoparticles (MIL-53, MIL-88A, MIL-100, and MIL-89) exhibit tunable pore sizes, structural flexibility, and demonstrated biocompatibility.^11^,^12^ MIL-89 offers distinct advantages over conventional nanocarriers, including liposomes, polymeric nanoparticles, and other MOFs, demonstrating superior drug loading capacity, enhanced structural adaptability, and more favorable physiological degradation kinetics.^13^ Horcajada et al reported that iron carboxylate MOFs like MIL-89 achieve significantly higher encapsulation efficiencies than polymeric or liposomal systems, with minimized burst release and capacity for both hydrophilic and hydrophobic drug entrapment.^13^

Our previous work established that nanoMIL-89 is effectively internalized by vascular endothelial and smooth muscle cells, localizes in endocytic vesicles, and maintains intracellular persistence across cell generations.^7^ The platform demonstrated excellent biocompatibility and anti-inflammatory properties, positioning it as an ideal candidate for cardiovascular therapeutics. Building on this foundation, we developed sildenafil-loaded nanoMIL-89 (Sil@nanoMIL-89), which exhibited sustained drug release over 72 hours, produced delayed vasodilatory responses in ex vivo aortic segments, and showed favorable in vitro safety profiles.^14^ This represented the first MOF-based delivery system evaluated for PAH treatment, establishing a critical proof-of-concept.

The current study advances this research by evaluating Bosentan-loaded nanoMIL-89 (Bos@nanoMIL-89) for the treatment of PAH. Considering Bosentan’s clinical importance coupled with its solubility challenges and dose-limiting side effects, we postulated that nanoMIL-89 encapsulation would: (1) enhance therapeutic efficacy; (2) mitigate inflammation and oxidative stress; and (3) promote vascular repair while maintaining cellular safety. We systematically assessed the cytotoxicity and therapeutic potential of Bos@nanoMIL-89 in HUVECs and HPASMCs under inflammatory conditions, evaluating critical parameters including cell viability, ROS generation, CXCL8 expression, genotoxicity, angiogenic capacity, and endothelin-1 regulation.

## Materials and Methods

### Synthesis and Physicochemical Characterization of NanoMIL-89

NanoMIL-89 nanoparticles were synthesized using a previously described hydrothermal method.^7^,^10^ Briefly, iron(III) chloride hexahydrate (FeCl₃·6H_2_O, 1 mmol; MW = 270.3; Honeywell, 10025–77-1) and trans–trans muconic acid (1 mmol; MW = 142.1; Aldrich, M90003-10G) were dissolved in absolute ethanol and glacial acetic acid (VWR, 20104.334). The reaction mixture was incubated at 90 °C for approximately 28 hours. The resulting precipitate was collected by centrifugation, washed sequentially with distilled water and ethanol, and air-dried overnight to yield a brown nanoMIL-89 powder.

Nanoparticle morphology and size were assessed using transmission electron microscopy (TEM) and scanning electron microscopy (SEM). Structural features were characterized by powder X-ray diffraction (PXRD), and functional groups were confirmed using Fourier-transform infrared spectroscopy (FTIR). The characterized nanoMIL-89 particles were subsequently used for Bosentan encapsulation and biological evaluation. All experiments were conducted using three independently synthesized batches.

### Bosentan Loading and Drug Release Profiling

Bosentan loading into nanoMIL-89 (Bos@nanoMIL-89) was performed as previously described.^10^ A stock solution of Bosentan (50 mg/mL) was prepared by dissolving 50 mg of Bosentan hydrate (Sigma-Aldrich, SML1265-50MG) in 1 mL of dimethyl sulfoxide (DMSO; Sigma-Aldrich, 34869–100ML; purity 99.7%). A working solution (0.5 mg/mL) was obtained by diluting 200 µL of the stock into 19.8 mL of a 1:1 mixture of DMSO and phosphate-buffered saline (PBS, pH 7.4). For encapsulation, 200 mg of nanoMIL-89 was added to 5 mL of the working solution and incubated at room temperature on a shaker for 16–18 hours.

The resulting Bos@nanoMIL-89 was collected by centrifugation (7,000 rpm, 15 minutes), and the supernatant was stored at −20 °C for subsequent analysis. The pellet was resuspended in fresh PBS containing 0.1% DMSO and centrifuged at various time points (0, 0.5, 1, 3, 6, 24, 48, 72, and 96 hours) to monitor release kinetics. In this experiment, nine technical replicates from three independently synthesized batches were used. Bosentan concentrations in the supernatants were quantified by high-performance liquid chromatography (HPLC), and loading efficiency was calculated accordingly.

### Cell Culture and Treatment

Human umbilical vein endothelial cells (HUVECs) and human pulmonary artery smooth muscle cells (HPASMCs) were obtained from Gibco (C0035C and C0075C, respectively) and cultured according to the manufacturer’s instructions. HUVECs were maintained in EGM-2 medium (PromoCell, C-22011), and HPASMCs in high-glucose DMEM (Capricorn, DMEM-H-12), both supplemented with 10% fetal bovine serum (FBS; ScienCell, 0500), 1% penicillin/streptomycin (Gibco, 15140122), and 1% endothelial cell growth supplement for HUVECs only (PromoCell, C-39215). Cells were incubated at 37 °C in a humidified atmosphere containing 5% CO_2_.

Cells were seeded at 10,000 cells per well in 96-well plates and allowed to adhere for 24 hours prior to treatment. Treatments included Bosentan (0.03125–0.5 mg/mL; Sigma-Aldrich, SML1265-50MG), nanoMIL-89 (0–100 µg/mL), and Bos@nanoMIL-89 (0–100 µg/mL), in the presence or absence of lipopolysaccharide (LPS, 1 µg/mL; Sigma-Aldrich, L2630). After 24 hours of exposure, cells and supernatants were collected for downstream analyses. All in vitro experiments were performed using nine technical replicates from three independently synthesized batches.

### Cell Viability and Cytotoxicity Assays

Cell viability was assessed using the AlamarBlue Cell Viability Reagent (Thermo Fisher Scientific, DAL1025) according to the manufacturer’s instructions. After treatment, a 10% AlamarBlue solution was added to each well, and cells were incubated at 37 °C for 4–6 hours. Fluorescence was measured using a Multiskan Sky microplate reader (Thermo Fisher Scientific) at an excitation wavelength of 570 nm and an emission wavelength of 600 nm.

Lactate dehydrogenase (LDH) release into the culture medium was quantified using the LDH Cytotoxicity Assay Kit (Abcam, ab183367). Briefly, 25 μL of LDH reaction mix was added to the conditioned media and incubated for 30 minutes. A stop solution was then added, and absorbance was recorded at 490 nm and 680 nm.

### Quantification of CXCL-8 and ET-1 by ELISA

CXCL-8 levels in cell supernatants were measured using the Human CXCL-8 ELISA Kit (R&D Systems, DY992). Samples from both basal and LPS-stimulated conditions were added to pre-coated wells, followed by incubation with a polyclonal antibody and substrate. Absorbance was measured at 450–570 nm, and cytokine concentrations were calculated using a standard curve.

Endothelin-1 (ET-1) was quantified using the Human Endothelin-1 ELISA Kit (Abcam, ab133030). Treated cell supernatants were processed according to the manufacturer’s protocol, and absorbance was measured at 450–570 nm using a Cytation 5 microplate reader (BioTek). Results are representative of three independent experiments.

### Assessment of Genotoxicity by Comet Assay

Genotoxicity was assessed using the OxiSelect™ Comet Assay Kit (Cell Biolabs, STA-351). HUVECs and HPASMCs were seeded in 12-well plates and treated with Bosentan, nanoMIL-89, or Bos@nanoMIL-89 for 24 hours. Cells were harvested, mixed with comet agarose, and applied to pre-coated slides. Following cell lysis and alkaline unwinding, electrophoresis was performed. DNA damage was visualized using Vista Green DNA Dye under a fluorescence microscope. Images were analyzed with ImageJ software (National Institutes of Health).

### Reactive Oxygen Species (ROS) Detection by DHE Assay

ROS levels in HUVECs were assessed using the Dihydroethidium (DHE) Assay Kit (Abcam, ab236206). Following treatment, the cells were washed with PBS and incubated with 10 μM DHE in fresh medium for 30 minutes at 37 °C. Fluorescence was measured using a Cytation 5 microplate reader (BioTek) at an excitation wavelength of 480–520 nm and an emission wavelength of 570–600 nm.

### Angiogenesis and Tube Formation Assay Using 3D Co-Culture

A 3D co-culture system was used to evaluate the angiogenic potential of Bos@nanoMIL-89. HPASMCs were seeded in 96-well plates and cultured for 24 hours, followed by the seeding of HUVECs on top. Treatments were applied one day later, and the co-culture was maintained in EGM-2 medium (PromoCell, C-22011) for 7 days, with medium changes every 2 days.

Endothelial tube formation was assessed by immunofluorescence staining using a primary anti-CD31 antibody (1:300; R&D Systems, AF806) and an Alexa Fluor 488-conjugated secondary antibody (1:200; Invitrogen, A11029). Nuclei were stained with Hoechst 33342 (Invitrogen, H3570), and cell membranes were labeled using a deep red cell mask (Invitrogen, C10046). Imaging was performed using an Operetta High-Content Imaging System (PerkinElmer) at 10× magnification. Tube formation parameters (eg, total length, number of junctions) were quantified using the Angiogenesis Analyzer plugin in ImageJ^15^ (National Institutes of Health), and cell counts were analyzed using Harmony software (PerkinElmer).

### Statistical Analysis

All experiments were conducted in triplicate unless otherwise stated. Data were analyzed using GraphPad Prism v9.2.0 (GraphPad Software, USA). Two-way ANOVA followed by Bonferroni post hoc tests was used to assess the effects of treatment concentrations across conditions. One-way ANOVA followed by Dunnett’s post hoc tests was applied to compare treatment groups (Bosentan alone, nanoMIL-89, Bos@nanoMIL-89) with the control. Data are presented as mean ± SEM, and p-values < 0.05 were considered statistically significant.

## Results

### Synthesis and Characterization of nanoMIL-89

**Figure 1:**
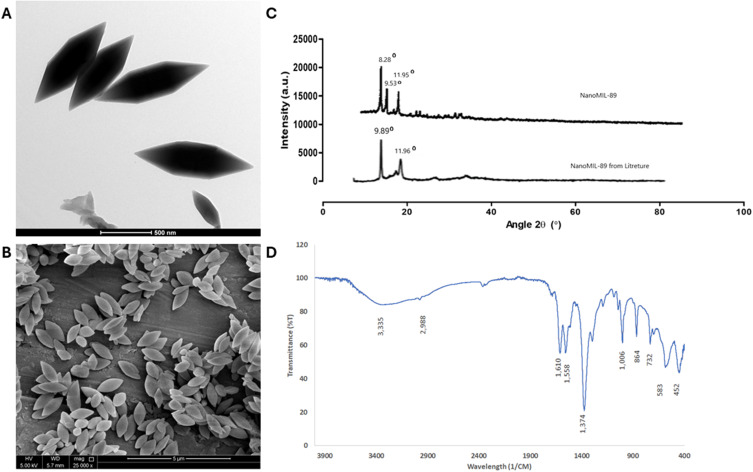
Physicochemical characterization of nanoMIL-89. (**A**) Transmission electron microscopy (TEM) image (50,000× magnification) showing hexagonal nanoparticle morphology. (**B**) Scanning electron microscopy (SEM) image (25,000× magnification) confirming uniform particle size and distribution. (**C**) Powder X-ray diffraction (PXRD) pattern indicating high crystallinity, consistent with the MIL-89 framework. (**D**) Fourier-transform infrared (FTIR) spectrum showing characteristic functional groups, confirming the chemical composition.

NanoMIL-89 nanoparticles were prepared by hydrothermal synthesis, and their morphological and structural properties were characterized. TEM and SEM images revealed hexagonal particles with an average width of 96 nm, observed in three independent batches (n = 15), without any aggregation (Figures 1A and 1B). These results are consistent with the uniformity and stability of the synthesized nanoMIL-89 particles, consistent with previous reports.^11^,^12^

PXRD pattern showed sharp diffraction peaks at 2θ values of 8.073°, 9.434°, and 11.895°, which are in agreement with the highly crystalline structure of MIL-89 (Figure 1C) and thus confirm the preservation of the MOF framework.^12^ FTIR spectroscopy also confirmed the chemical identity of the synthesized material, showing characteristic peaks at ~1610 cm^−^¹ (Sharp peak): C=C stretching (of trans,trans-muconate linker), ~1558 cm^−^¹ is COO^−^ stretching (carboxylate group coordinated to Fe), ~1374 cm^−^¹ is Symmetric COO‾ stretching (carboxylate-Fe coordination), confirms binding of linker to metal, ~1006 cm^−^¹ is C-O stretching (from linker or residual ethanol), 500–700 cm^−^¹ (Multiple bands) is Fe-O vibrations (metal-oxygen coordination) confirms the formation of Fe^3^⁺ node (Figure 1D). The absence of unexpected peaks indicates high chemical purity and structural stability.

These results confirm the successful synthesis of crystalline, chemically stable nanoMIL-89 particles, which are suitable for drug encapsulation and further biomedical applications.

### Bosentan Release Profile From nanoMIL-89

The release kinetics of Bosentan from nanoMIL-89 (Bos@nanoMIL-89) were evaluated using HPLC over a 96-hour period. The biphasic release profile consisted of an initial burst phase, during which approximately 1,500 ng/mL of Bosentan was released within the first hour, followed by a sustained release phase with concentrations stabilizing around 500 ng/mL. Sampling was performed at defined time points (0, 0.5, 1, 3, 6, 24, 48, 72, and 96 hours) to capture both the rapid and prolonged release phases. The loading efficiency was calculated to be approximately 97%. This dual-phase release pattern suggests initial surface desorption followed by diffusion of Bosentan from the internal MOF matrix, demonstrating the system’s capacity for controlled drug delivery.

**Figure 2:**
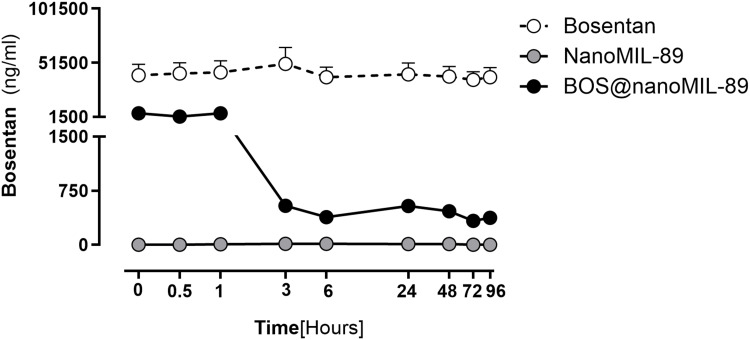
Bosentan release profile from nanoMIL-89 (Bos@nanoMIL-89) assessed by HPLC. A biphasic release pattern was observed, consisting of an initial burst phase followed by sustained release over 96 hours. Free Bosentan was used as a positive control, exhibiting immediate and continuous release, while unloaded nanoMIL-89 served as a negative control with no detectable drug release. The Y-axis is split (0–1500 ng/mL and 51,500–101,500 ng/mL) to facilitate visualization of both low and high concentration ranges.

Two controls were prepared to validate the specificity of the formulation. Free Bosentan maintained a constant concentration of approximately 50,000 ng/mL throughout the experiment because it immediately dissolved and diffused quickly in the release medium. The negative control containing nanoMIL-89 did not show any detectable Bosentan release, which proved that the MOF structure remains inert when it is not loaded with the drug. The results demonstrate that nanoMIL-89 functions as a sustained drug delivery system that regulates Bosentan release patterns throughout time (Figure 2).

### Effect of BOS@nanoMIL-89 Treatment on Cellular Viability

**Figure 3:**
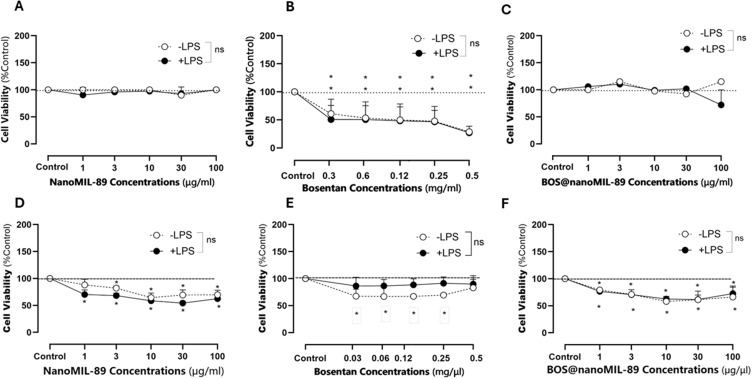
Effect of nanoMIL-89, Bosentan, and Bos@nanoMIL-89 on cell viability in HUVECs and HPASMCs under normal (–LPS) and inflammatory (+LPS) conditions. (**A–C**) Viability of HUVECs treated with nanoMIL-89 (**A**), Bosentan (**B**), or Bos@nanoMIL-89 (**C**). (**D–F**) Viability of HPASMCs treated with nanoMIL-89 (**D**), Bosentan (**E**), or Bos@nanoMIL-89 (**F**). Statistical analysis was performed using two-way ANOVA with Bonferroni post-hoc test and Dunnett’s multiple comparisons versus control. Error bars represent mean ± SEM (n = 3). *p < 0.05; ns = not significant.

The effect of nanoMIL-89, free Bosentan, and Bos@nanoMIL-89 on cellular viability was evaluated in HUVECs and HPASMCs under basal and inflammatory conditions (±LPS). NanoMIL-89 or Bos@nanoMIL-89 at all tested concentrations (1–100 µg/mL) had no significant effect on cell viability in HUVECs, regardless of LPS stimulation (Figures 3A and 3C). Bosentan alone did not cause a significant decrease in HUVEC viability except at the highest concentrations tested, where a slight but statistically significant reduction was observed (Figure 3B).

HPASMCs showed a clear dose-dependent anti-proliferative response to Bosentan and Bos@nanoMIL-89 in the presence and absence of LPS (Figures 3E and 3F). A modest reduction in cell viability was also observed following nanoMIL-89 treatment at higher doses (≥30 µg/mL; Figure 3D). These findings suggest that Bos@nanoMIL-89 and nanoMIL-89 are well tolerated by endothelial cells, but may exert selective anti-proliferative effects on smooth muscle cells, which may help to reduce vascular remodeling in the PAH setting.

### Cellular Cytotoxicity Assessment of BOS@nanoMIL-89 Treatment

**Figure 4:**
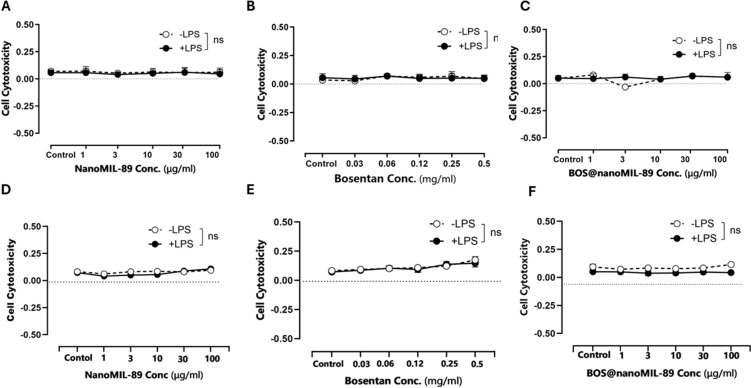
Cytotoxicity analysis of nanoMIL-89, Bosentan, and Bos@nanoMIL-89 in HUVECs and HPASMCs under normal (–LPS) and inflammatory (+LPS) conditions using the LDH assay. (**A–C**) Cytotoxicity profiles in HUVECs treated with nanoMIL-89 (**A**), Bosentan (**B**), and Bos@nanoMIL-89 (**C**). (**D–F**) Cytotoxicity profiles in HPASMCs treated with nanoMIL-89 (**D**), Bosentan (**E**), and Bos@nanoMIL-89 (**F**). Statistical analysis was performed using a two-way ANOVA followed by a Bonferroni post-hoc test and Dunnett’s multiple comparisons test versus the control. Error bars represent mean ± SEM (n = 3); *p < 0.05; ns = not significant.

The lactate dehydrogenase (LDH) release assay was used to measure the cytotoxic effects of nanoMIL-89, free Bosentan, and Bos@nanoMIL-89 in HUVECs and HPASMCs under both basal (–LPS) and inflammatory (+LPS) conditions. NanoMIL-89 and Bos@nanoMIL-89 showed low cytotoxic effects at all concentrations from 1 to 100 µg/mL because they did not produce significant differences compared to untreated controls in HUVECs and HPASMCs (Figures 4A, 4C, 4D, and 4F). The results confirm that both untreated and drug-loaded MOF formulations maintain good compatibility with biological systems.

Free Bosentan treatment showed a small increase in LDH release at concentrations above 0.25 mg/mL, especially in HPASMCs (Figures 4B and 4E). The observed trend lacked statistical significance, yet remained within the non-toxic range, indicating that Bosentan at the tested concentrations does not cause major cytotoxic damage. The data show that Bos@nanoMIL-89 and its carrier maintain excellent cellular tolerance because they do not damage membrane integrity in either physiological or inflammatory conditions (Figure 4).

### CXCL8 Inflammatory Cytokine Response Following Bos@nanoMIL-89 Treatment

These findings indicate that Bos@nanoMIL-89 integrates the anti-inflammatory activity of Bosentan with the nanocarrier’s enhanced delivery properties, resulting in a pronounced reduction of CXCL8 secretion in both endothelial and smooth muscle cells during inflammation.

**Figure 5:**
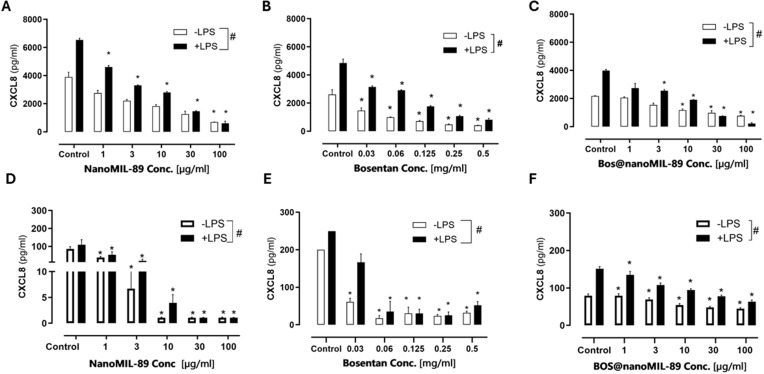
Effect of nanoMIL-89, Bosentan, and Bos@nanoMIL-89 on CXCL8/IL-8 cytokine release in HUVECs and HPASMCs under normal (–LPS) and inflammatory (+LPS) conditions. (**A–C**) CXCL8 levels in HUVECs after treatment with nanoMIL-89 (**A**), Bosentan (**B**), and Bos@nanoMIL-89 (**C**). (**D–F**) CXCL8 levels in HPASMCs after treatment with nanoMIL-89 (**D**), Bosentan (**E**), and Bos@nanoMIL-89 (**F**). Statistical analysis was performed using two-way ANOVA with Bonferroni correction and one-way ANOVA with Dunnett’s multiple comparisons versus control. Error bars represent mean ± SEM (n = 3); *p < 0.05 vs control; #p < 0.05 between +LPS and –LPS conditions.

The anti-inflammatory effects of Bos@nanoMIL-89 were assessed by quantifying interleukin-8 (CXCL8/IL-8) secretion from HUVECs and HPASMCs using ELISA under both basal (–LPS) and inflammatory (+LPS) conditions. All treatments (nanoMIL-89, free Bosentan, and Bos@nanoMIL-89) demonstrated dose-dependent reductions in CXCL8 release (Figure 5).

In HUVECs, nanoMIL-89 and Bos@nanoMIL-89 significantly suppressed CXCL8 secretion at concentrations ≥10 µg/mL under both conditions (Figures 5A and 5C). Free Bosentan showed comparable inhibition, reaching statistical significance at ≥0.06 mg/mL (Figure 5B). HPASMCs exhibited similar dose-responsive decreases, with significant reductions observed from 10 µg/mL onward for both nanoMIL-89 and Bos@nanoMIL-89 (Figures 5D–5F).

Quantitative analysis revealed maximal CXCL8 suppression of 64.38% (HUVECs) and 43.34% (HPASMCs) under basal conditions. During LPS-induced inflammation, inhibition increased substantially to 94.20% (HUVECs) and 58.14% (HPASMCs), demonstrating potent anti-inflammatory activity.

These results demonstrate that Bos@nanoMIL-89 integrates Bosentan’s pharmacological activity with the delivery advantages of the nanocarrier, resulting in enhanced suppression of CXCL8 secretion in both endothelial and smooth muscle cells during inflammation., particularly under inflammatory conditions. (see Supplementary Table S1 for complete numerical values).

### Reduction of Endothelin-1 (ET-1) Secretion by Bos@nanoMIL-89

**Figure 6:**
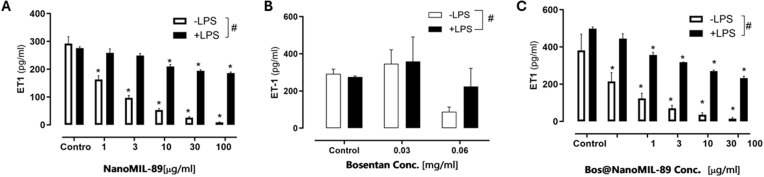
Effect of nanoMIL-89, Bosentan, and Bos@nanoMIL-89 on Endothelin-1 (ET-1) release in HUVECs under normal (–LPS) and inflammatory (+LPS) conditions. (**A**) NanoMIL-89 significantly reduced ET-1 secretion in a dose-dependent manner. (**B**) Free Bosentan showed no significant effect at the tested concentrations. (**C**) Bos@nanoMIL-89 markedly suppressed ET-1 levels across all doses tested. Statistical analysis was conducted using two-way ANOVA with Bonferroni post-hoc test and one-way ANOVA with Dunnett’s multiple comparisons versus the control. Error bars represent mean ± SEM (n = 3); *p < 0.05 vs control; #p < 0.05 for comparisons between LPS-treated and untreated groups.

The impact of nanoMIL-89, free Bosentan, and Bos@nanoMIL-89 on Endothelin-1 (ET-1) release was assessed in HUVECs by ELISA under both basal and LPS-stimulated conditions. NanoMIL-89 produced a significant dose-dependent reduction in ET-1 secretion, particularly at ≥10 µg/mL (Figure 6A). Bos@nanoMIL-89 elicited a statistically significant and even stronger decrease in ET-1 levels across all concentrations tested, with maximum inhibition observed at 30 and 100 µg/mL (Figure 6C).

Quantitatively, ET-1 levels were reduced by up to 96.68% under basal conditions and by 53.36% under inflammatory conditions. In contrast, free Bosentan at 0.03 and 0.06 mg/mL did not induce significant changes in ET-1 secretion (Figure 6B).

These results highlight the synergistic benefits of the Bos@nanoMIL-89 formulation in mitigating vasoconstrictive signaling in endothelial cells through enhanced ET-1 suppression. (see Supplementary Table S1 for complete numerical values).

### Genotoxicity Assessment of Bos@nanoMIL-89 in Vascular Cells

**Figure 7:**
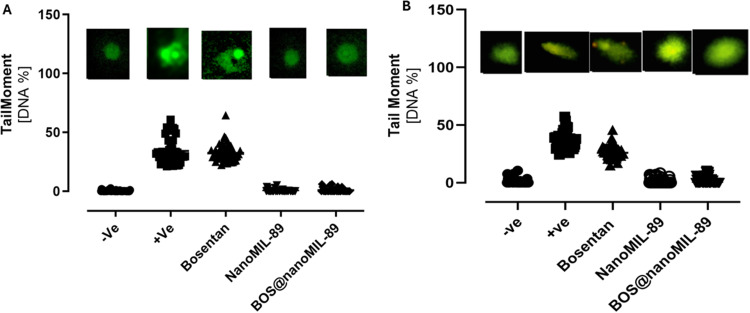
Genotoxicity assessment of Bosentan, nanoMIL-89, and Bos@nanoMIL-89 in HUVECs and HPASMCs using the comet assay (single-cell gel electrophoresis). (**A**) Representative comet images and quantification of tail moment (% DNA in the tail) in HUVECs. (**B**) Corresponding results for HPASMCs. Cells were treated with Bosentan (0.5 mg/mL), nanoMIL-89 (10 µg/mL), or Bos@nanoMIL-89 (10 µg/mL). Data represent tail moment measurements from 15 nuclei per group (n = 15).

The genotoxic effects of nanoMIL-89, Bosentan, and Bos@nanoMIL-89 were evaluated in HUVECs and HPASMCs using the comet assay analysis. The nanoMIL-89 or Bos@nanoMIL-89 solution, at a concentration of 10 μg/mL, or the free Bosentan solution, at a concentration of 0.5 mg/mL, was applied to the cells. The comet assay measured DNA damage by calculating the tail moment (% DNA in the tail), which indicates strand breakage (Figure 7).

Free Bosentan treatment resulted in substantial DNA fragmentation in both cell types, as evidenced by the production of longer comet tails and higher tail moments. The tail moment values of the nanoMIL-89 and Bos@nanoMIL-89 samples matched those of the negative control and remained significantly lower than those of the positive control.

The results demonstrate that nanoMIL-89 and Bos@nanoMIL-89 do not cause detectable DNA damage in either vascular endothelial or smooth muscle cells when used alone or in combination. These findings support the genomic safety profile of the nanoformulation and also demonstrate its biocompatibility in vitro.

### Suppression of Reactive Oxygen Species (ROS) Formation by Bos@nanoMIL-89

**Figure 8:**
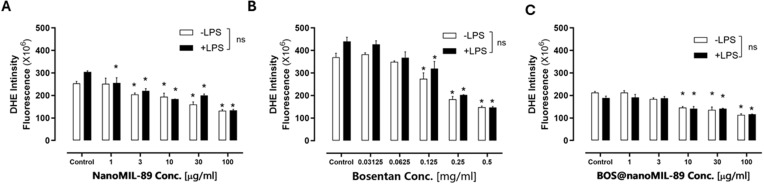
Effect of nanoMIL-89, Bosentan, and Bos@nanoMIL-89 on ROS generation in HUVECs under normal (–LPS) and inflammatory (+LPS) conditions, assessed by the DHE fluorescence assay. (**A**) NanoMIL-89 significantly decreased DHE fluorescence intensity in a dose-dependent manner. (**B**) Free Bosentan reduced ROS levels at higher concentrations. (**C**) Bos@nanoMIL-89 consistently suppressed ROS levels across all tested concentrations. Statistical analysis was performed using two-way ANOVA with Bonferroni post-hoc tests and one-way ANOVA with Dunnett’s multiple comparisons versus control. Data are presented as mean ± SEM (n = 3); *p < 0.05 vs control.

The antioxidant effects of Bos@nanoMIL-89, nanoMIL-89, and free Bosentan were evaluated in HUVECs by measuring intracellular superoxide levels using the DHE fluorescence assay under both basal (–LPS) and inflammatory (+LPS) conditions. All three treatments resulted in a dose-dependent reduction in ROS levels, with a maximum decrease of approximately 46.28% under basal conditions and 38.19% under inflammatory conditions (Figure 8).

NanoMIL-89 alone significantly reduced ROS levels at concentrations ≥3 µg/mL, with comparable effects observed in both media conditions (Figure 8A). Free Bosentan also demonstrated antioxidant activity, significantly lowering DHE fluorescence at concentrations ≥0.125 mg/mL (Figure 8B). Notably, Bos@nanoMIL-89 exhibited the most consistent and potent ROS suppression across all tested concentrations (Figure 8C), indicating a superior oxidative stress-modulating effect compared to its individual components.

These findings suggest that Bos@nanoMIL-89 functions not only as a drug delivery system but also as an active nanotherapeutic capable of reducing oxidative stress in endothelial cells. (see Supplementary Table S1 for complete numerical values).

### In vitro Angiogenesis and Endothelial Network Formation

**Figure 9:**
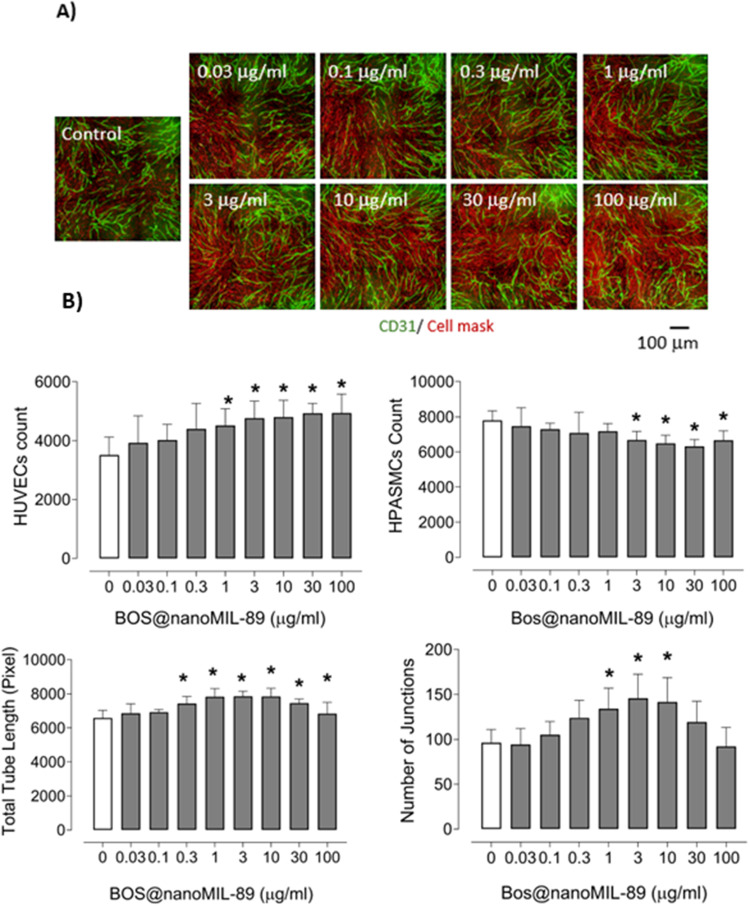
Angiogenic effects of Bos@nanoMIL-89 in a 3D co-culture model of HUVECs and HPASMCs. (**A**) Representative immunofluorescence images showing dose-dependent enhancement of endothelial tube formation. Endothelial cells were stained with anti-CD31 (green), and all cell membranes were labeled using a red cell mask; scale bar, 100 μm. (**B**) Quantitative analysis of endothelial and smooth muscle cell counts, total tube length, and number of junctions across increasing concentrations of Bos@nanoMIL-89. Nine fields were analyzed per well (n = 6–12 per condition). Data are presented as mean ± SEM. Statistical analysis was performed using one-way ANOVA; *p < 0.05 vs control.

The pro-angiogenic activity of Bos@nanoMIL-89 was evaluated using a 3D co-culture model of HUVECs and HPASMCs. The formation of endothelial tubes and vascular network morphology was evaluated by CD31 immunostaining, and the abundance of endothelial and smooth muscle cells, total tube length, and the number of junctions were quantified (Figure 9).

Fluorescent imaging showed a dose-dependent increase in CD31+ endothelial structures with increasing concentrations of Bos@nanoMIL-89, especially at 3 and 10 µg/mL (Figure 9A). Quantitative analysis revealed that total tube length and number of junctions increased at these concentrations, but decreased or remained constant at 30 and 100 µg/mL (Figure 9B). HUVEC counts increased across all doses, while HPASMC numbers decreased, suggesting a shift favoring endothelial cell growth and angiogenesis while suppressing smooth muscle proliferation—a desirable therapeutic effect in PAH.

**Figure 10:**
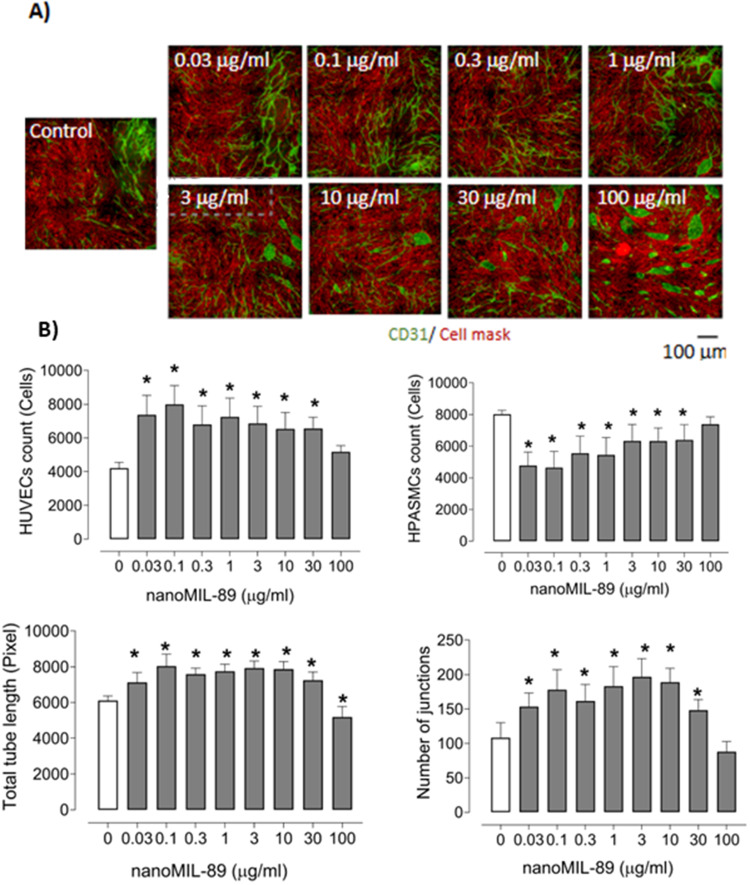
Angiogenic effects of nanoMIL-89 at various concentrations in a 3D co-culture model of HUVECs and HPASMCs. (**A**) Representative immunofluorescence images showing dose-dependent changes in endothelial network formation. Endothelial cells were stained with an anti-CD31 antibody (green), and cell membranes were labeled with a red cell mask; scale bar: 100 μm. (**B**) Quantitative analysis of HUVEC and HPASMC counts, total tube length, and number of junctions following nanoMIL-89 treatment. Nine fields were analyzed per well (n = 6–12 per condition). Data are presented as mean ± SEM. Statistical comparisons were performed using one-way ANOVA; *p < 0.05 vs control.

The angiogenic effects of nanoMIL-89 and free Bosentan were evaluated separately. NanoMIL-89 alone treatment resulted in a significant increase in total tube length and junction formation at concentrations up to 30 µg/mL. However, endothelial clustering was observed at 100 µg/mL, indicating a loss of organized tube structure (Figure 10). This effect was accompanied by an increase in endothelial cell count and a reduction in smooth muscle cell numbers.

**Figure 11:**
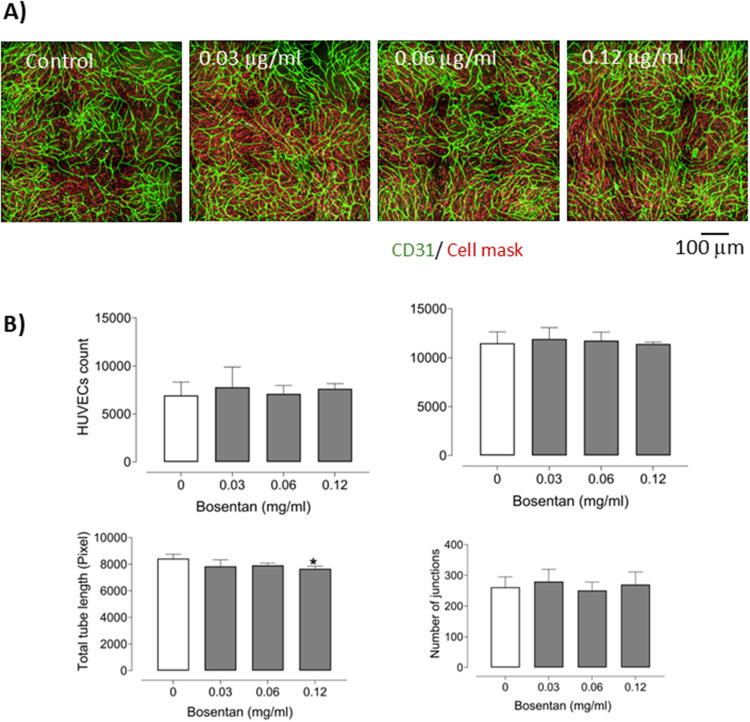
Angiogenic effects of free Bosentan at various concentrations in a 3D co-culture model of HUVECs and HPASMCs. (**A**) Representative immunofluorescence images showing endothelial network formation following Bosentan treatment. Endothelial structures were stained with anti-CD31 antibody (green), and cell membranes were labeled with a red cell mask; scale bar: 100 μm. (**B**) Quantitative analysis of HUVEC and HPASMC counts, total tube length, and number of junctions after treatment with increasing doses of Bosentan. Nine fields were quantified per well (n = 6–12 per condition). Data are presented as mean ± SEM. Statistical analysis was performed using one-way ANOVA; *p < 0.05 versus control.

Free Bosentan also exhibited modest angiogenic activity. A significant increase in total tube length was observed at 0.125 mg/mL, while the number of junctions remained unchanged across all tested doses. No significant changes were detected in HUVEC or HPASMC counts compared to controls (Figure 11).

These results confirm that Bos@nanoMIL-89 has superior angiogenic activity compared to its components. It promotes endothelial network formation while suppressing smooth muscle proliferation. The observed effects suggest that it may have a role in reversing vascular remodeling in PAH.

## Discussion

PAH is a severe and life-threatening vascular disease characterized by an increase in pulmonary vascular resistance, linked to endothelial dysfunction, inflammation, and pathological remodeling of the pulmonary arteries. These changes are sustained by hyperproliferative SMCs, defective endothelial repair, oxidative stress, and disturbed inflammatory responses. Although currently available pharmacotherapies, for instance, the endothelin receptor antagonist Bosentan, are useful in clinical practice, their application is hampered by systemic toxicity, short plasma half-life, and poor target specificity. These challenges underscore the need for advanced drug delivery systems that can simultaneously interact with multiple pathophysiological mechanisms of PAH.

In this study, we explored the therapeutic efficacy of Bosentan encapsulated in nanoMIL-89 (Bos@nanoMIL-89), a newly synthesized metal–organic framework (MOF) nanocarrier, in two major vascular cell lines, HUVECs and HPASMCs, under both normal and inflammatory conditions. Based on our earlier research that established the safety and cellular uptake of nanoMIL-89 as well as its in vivo tolerability in zebrafish embryos, we here provide the first extensive in vitro assessment of the biological effects of Bos@nanoMIL-89 on cell viability, inflammation, oxidative stress, genotoxicity, and angiogenesis.^4^,^6^

Bos@nanoMIL-89 demonstrated good biocompatibility and did not induce cytotoxicity in either cell line. Notably, while free Bosentan exhibited a dose-dependent cytotoxic effect on HPASMCs, this effect was reversed when the drug was encapsulated in the MOF, possibly due to the MOF’s controlled delivery properties. Importantly, Bos@nanoMIL-89 inhibited the proliferation of HPASMCs while preserving the viability of endothelial cells, which is crucial in PAH, as excessive smooth muscle cell proliferation leads to vessel blockage and remodeling.^16^,^17^

In addition to its antiproliferative effects, Bos@nanoMIL-89 showed pronounced anti-inflammatory activity. This suggests that Bosentan and the MOF may have a synergistic anti-inflammatory effect, as evidenced by the decreased levels of CXCL8 (IL-8) in both HUVECs and HPASMCs treated with LPS. This agrees with earlier reports, which have proposed that the anti-inflammatory properties of MIL-89 may be due to its iron content and its ability to modulate cellular signaling pathways.^4^

Additionally, in line with its therapeutic potential, Bos@nanoMIL-89 resulted in a substantial decrease in endothelin-1 (ET-1) levels, a key mediator of vasoconstriction and vascular remodeling in PAH. Notably, this was not observed with free Bosentan, thus suggesting that the nanoformulation might have better pharmacokinetic and pharmacodynamic properties. This finding also demonstrates that MOF-based carriers can enhance the bioavailability of encapsulated drugs within cells.

Genotoxicity assays showed that nanoMIL-89 has a protective role. On the other hand, Bos@nanoMIL-89 and the carrier alone showed no significant DNA fragmentation, while free Bosentan had the most pronounced effect. These data suggest that nanoMIL-89 may protect against DNA damage, which could be attributed to its antioxidant effects or its ability to influence DNA repair mechanisms.^18–20^ Notably, the genotoxicity assay was conducted using a 0.5 mg/mL concentration of free Bosentan, which is substantially higher than the typical therapeutic plasma concentration (1–3 µg/mL) observed in patients due to extensive first-pass metabolism. This supraphysiological concentration was selected to model high-dose stress conditions and evaluate potential genotoxic liabilities. Under these conditions, Bos@nanoMIL-89 exhibited significantly reduced DNA fragmentation compared to free Bosentan, highlighting the protective effect of the MOF carrier, likely through controlled release and reduced intracellular accumulation.

ROS production was significantly decreased in both cell lines when treated with Bos@nanoMIL-89. Given the importance of oxidative stress in the pathogenesis of endothelial dysfunction and vascular remodeling in PAH, this antioxidant capability is a significant advantage to the formulation.^21^,^22^

We also assessed the angiogenic capacity of Bos@nanoMIL-89 in a co-culture system. At intermediate concentrations, the formulation significantly enhanced endothelial tube formation, improved cell junctions, and decreased HPASMC density, thereby exhibiting dual effects that promote vascular regeneration and reduce smooth muscle-driven remodeling.^23^

Although the in vitro results of Bos@nanoMIL-89 are promising, it is essential to consider these results within the context of existing PAH treatment options. The current therapies, including Bosentan, Ambrisentan, Sildenafil, and prostacyclin analogs, primarily focus on vasodilation or inflammation; however, they have several disadvantages, such as side effects, frequent administration, and low efficacy. Bos@nanoMIL-89, on the other hand, is a single, multifunctional platform that targets various disease drivers, including proliferation, inflammation, oxidative stress, and angiogenesis.

From a nanotechnology perspective, other carriers, including liposomes, polymeric nanoparticles, and other MOFs (such as ZIF-8 and UiO-66), have been investigated for the pulmonary delivery of drugs. In comparison with other systems, nanoMIL-89 offers several benefits, including a high surface area, adjustable porosity, degradability, and bioactivity. Its iron-based framework may provide some extra therapeutic benefits, such as antioxidant and anti-inflammatory properties, which are not usually seen with non-active carriers. Furthermore, the results of this study indicate that nanoMIL-89 exhibits better cellular uptake and bioactivity than free Bosentan, lower genotoxicity, and a more favorable safety profile. Further research is needed to establish the effectiveness of Bos@nanoMIL-89 against other PAH drug nanoformulations in terms of pharmacokinetics and biodistribution, as well as its therapeutic index.

Although these findings are promising, the current research is based solely on short-term in vitro experiments. Although the LPS-induced inflammatory model is relevant, it does not completely mimic the complex pathophysiology of PAH in vivo. Additionally, the intracellular signaling pathways affected by Bos@nanoMIL-89, including NF-κB, MAPK, and eNOS, are not well understood. Future studies should also investigate the mechanisms of action, long-term safety, biodistribution, and efficacy of Bos@nanoMIL-89 in animal models. The surface of the MOF can be further modified with targeting ligands to increase vascular specificity for delivery to diseased pulmonary tissue.

In sum, this study has established that Bos@nanoMIL-89 is a safe nanotherapeutic platform with the potential to modulate multiple pathophysiological processes relevant to PAH. This formulation has the potential for future development as an advanced therapeutic option for pulmonary vascular diseases, as it targets endothelial dysfunction, inflammation, oxidative stress, and smooth muscle cell proliferation.

## Conclusion

This study provides an extensive in vitro assessment of Bosentan-loaded MIL-89 nanoparticles (Bos@nanoMIL-89) as a new nanomedicine for the treatment of pulmonary arterial hypertension (PAH). The successful synthesis and physicochemical characterization of the MOF-based system revealed high Bosentan loading efficiency and a biphasic drug release profile, which may offer improved release kinetics. While this could potentially translate to reduced dosing frequency and lower systemic toxicity, such benefits remain to be confirmed through dedicated in vivo pharmacokinetic and efficacy studies.

Bos@nanoMIL-89 exhibited multiple functional therapeutic effects, including selective inhibition of smooth muscle cell proliferation, preservation of endothelial viability, suppression of pro-inflammatory cytokine (CXCL8) and vasoconstrictor (ET-1) production, reduction of oxidative stress, and lack of significant genotoxicity. Additionally, it exhibits pro-angiogenic properties, which can aid in vascular regeneration, a significant unmet need in PAH treatment.

The formulation was found to have superior biological outcomes when compared to free Bosentan and nanoMIL-89 alone, indicating possible synergistic effects between the drug and the MOF carrier. Compared to current PAH pharmacotherapies and other nanocarrier systems, Bos@nanoMIL-89 offers unique advantages in drug release control, biocompatibility, and multifunctionality, making it a promising therapeutic nanomedicine.

However, this work still has some limitations, including the lack of in vivo experiments and a detailed analysis of the mechanisms. Further studies should include long-term safety, pharmacokinetics, biodistribution, and efficacy in animal models, as well as the intracellular signaling pathways modulated by the nanoformulation. Furthermore, long-term studies are warranted to evaluate the degradation behavior of MIL-89 and its potential impact on systemic iron homeostasis. While prior in vivo studies with sildenafil-loaded MIL-89 demonstrated favorable safety and biodistribution profiles, a comprehensive analysis of chronic exposure, iron accumulation, and biodegradation products remains essential for clinical translation.

In conclusion, Bos@nanoMIL-89 is a potent and versatile nanomedicine that can simultaneously address inflammation, oxidative stress, vascular remodeling, and endothelial dysfunction in PAH. These findings provide a basis for further development of this nano platform for preclinical testing and clinical application.

## Abbreviations

PAH, Pulmonary Arterial Hypertension; nanoMIL-89, iron-Based Metal–Organic Framework MIL-89; HUVECs, Human Umbilical Vein Endothelial Cells; HPASMCs, Human Pulmonary Artery Smooth Muscle Cells; ROS, Reactive Oxygen Species; ET-1, Endothelin-1; MOFs, Metal–Organic Frameworks; Sil@nanoMIL-89, Metal–Organic Frameworks; BOS@nanoMIL-89, Bosentan-loaded nanoMIL-89; TEM, Transmission Electron Microscopy; SEM, Scanning Electron Microscopy; PXRD, Powder X-ray Diffraction; FTIR, Fourier-Transform Infrared Spectroscopy; DMSO, Dimethyl Sulfoxide; HPLC, High-Performance Liquid Chromatography; EGM-2, Endothelial Cell Growth 2 media; LPS, Lipopolysaccharide; LDH, Lactate Dehydrogenase; DHE, Dihydroethidium; R%, Reduction Percentage.
